# mimicDetector: a pipeline for protein motif mimicry detection in host-pathogen interactions

**DOI:** 10.1093/bioinformatics/btag012

**Published:** 2026-01-12

**Authors:** Kaylee D Rich, James D Wasmuth

**Affiliations:** Faculty of Veterinary Medicine, University of Calgary, Calgary, Alberta, T2N 4Z6, Canada; Faculty of Veterinary Medicine, University of Calgary, Calgary, Alberta, T2N 4Z6, Canada

## Abstract

**Motivation:**

Molecular mimicry is used by pathogens to evade the host immune system and manipulate other host cellular processes. It is often mediated by short motifs in non-homologous proteins, whose detection challenges the sensitivity and specificity of existing bioinformatics tools.

**Results:**

We present mimicDetector, a *k*-mer-based pipeline for identifying protein-level molecular mimicry between pathogens and their hosts. Applied to 17 globally important pathogens, mimicDetector identified a broad and biologically plausible set of mimicry candidates, including helminth proteins mimicking components of the human complement system and a *Leishmania infantum* mimic of Reticulon-4, a regulator of immune cell recruitment.

**Availability and implementation:**

mimicDetector is freely available at https://github.com/kayleerich/mimicDetector/, implemented in Python and Snakemake, and compatible with Unix-based systems.

## 1 Introduction

Pathogens often evade immune detection through molecular mimicry, where their molecules structurally or functionally imitate those of the host. Mimicry has been described in glycans, lipids, and nucleic acids, with protein mimicry arguably the most widely studied (van Die and Cummings 2010, Buck *et al.* 2014, Bosch *et al.* 2021, Maguire *et al.* 2024). Identifying mimicry is important for understanding immune evasion, identifying therapeutic targets and revealing co-evolution.

Protein mimicry typically involves short motifs (5-to-20 amino acids in length), that mediate host-pathogen interactions by altering signaling, protein binding, or immune recognition [as reviewed by Sámano-Sánchez and Gibson (2020)]. However, these short motifs are difficult to detect computationally. Current state-of-the-art motif discovery tools, including those from the MEME suite or SLiMFinder, are designed to find overrepresented motifs among non-homologous, functionally related sequences (e.g. co-regulated genes) (Edwards *et al.* 2007, Bailey *et al.* 2015). Other tools, such as SLiMPred and SLiMDisc, predict motifs documented in curated databases like ELM (Mooney *et al.* 2012, Palopoli *et al.* 2015). These approaches work well for short linear motifs (SLiM) and post-translational modification (PTM) sites, but assume recurrence for prior motif knowledge. These assumptions do not hold for molecular mimicry, where motifs are typically rare, non-recurrent, and largely unknown. Consequently, many mimicry exploration studies rely on local-local sequence alignment tools despite their default parameters being poorly suited for short sequence fragments (Ludin *et al.* 2011, Doxey and McConkey 2013, Pearson 2013, Armijos-Jaramillo *et al.* 2021, Emiliani *et al.* 2022).

We previously resurrected a *k*-mer-based strategy that aligned pathogen-derived peptides against host and control proteomes (Ludin *et al.* 2011, Rich *et al.* 2023). While effective, it relied on strict identity thresholds that may exclude biologically relevant mimics with subtle sequence divergence. Additionally, homologue filtering—intended to reduce noise—risked discarding genuine mimics.

Here, we present mimicDetector, an optimized and scalable pipeline for identifying mimicry motifs across divergent proteomes without prior motif knowledge. Unlike existing motif-finding tools, mimicDetector addresses a distinct biological problem with different biological assumptions: detecting short, non-recurrent mimicry motifs that other algorithms are not designed to uncover. To achieve this, we systematically evaluated alignment algorithms, scoring matrices, homologue handling, and filtering strategies to improve sensitivity and specificity.

## 2 Benchmarking and optimization

### 2.1 Overall approach

To guide tool selection and parameter optimization for mimicDetector, we designed benchmarking experiments using identical-hit and similar-hit peptide datasets from three well-annotated, phylogenetically diverse reference proteomes used in prior mimicry studies: *Plasmodium falciparum* 3D7, *Mycobacterium tuberculosis* H37Rv, and *Brugia malayi* (Ludin *et al.* 2011, Muthye and Wasmuth 2023, Rich *et al.* 2023, Yanik *et al.* 2023). A full list of the proteomes used in this study is provided in Table S1, available as supplementary data at *Bioinformatics* online.

We evaluated the recall performance of five protein alignment search tools (BLASTP, DIAMOND, PHMMER, TOPAZ, and Glam2Scan), comparing substitution matrices (BLOSUM62 and PAM30), and, where possible, the *wordsize* (2 and 3) used for seeding the alignments (Fig. S1, available as supplementary data at *Bioinformatics* online) (Altschul *et al.* 1990, Frith *et al.* 2008, Eddy 2011, Buchfink *et al.* 2015, Medlar and Holm 2018). Peptides were extracted as overlapping *k*-mers (lengths 5-to-14), and 10 sets of 1000 *k*-mers were randomly sampled from the proteome (Li 2018). We used each tool and various parameter combinations to align these *k*-mers back to each original proteome (Table S2, available as supplementary data at *Bioinformatics* online), and used a custom script to measure the number of identical and similar *k*-mers, allowing up to *k*-3 mismatches.

**Figure 1:**
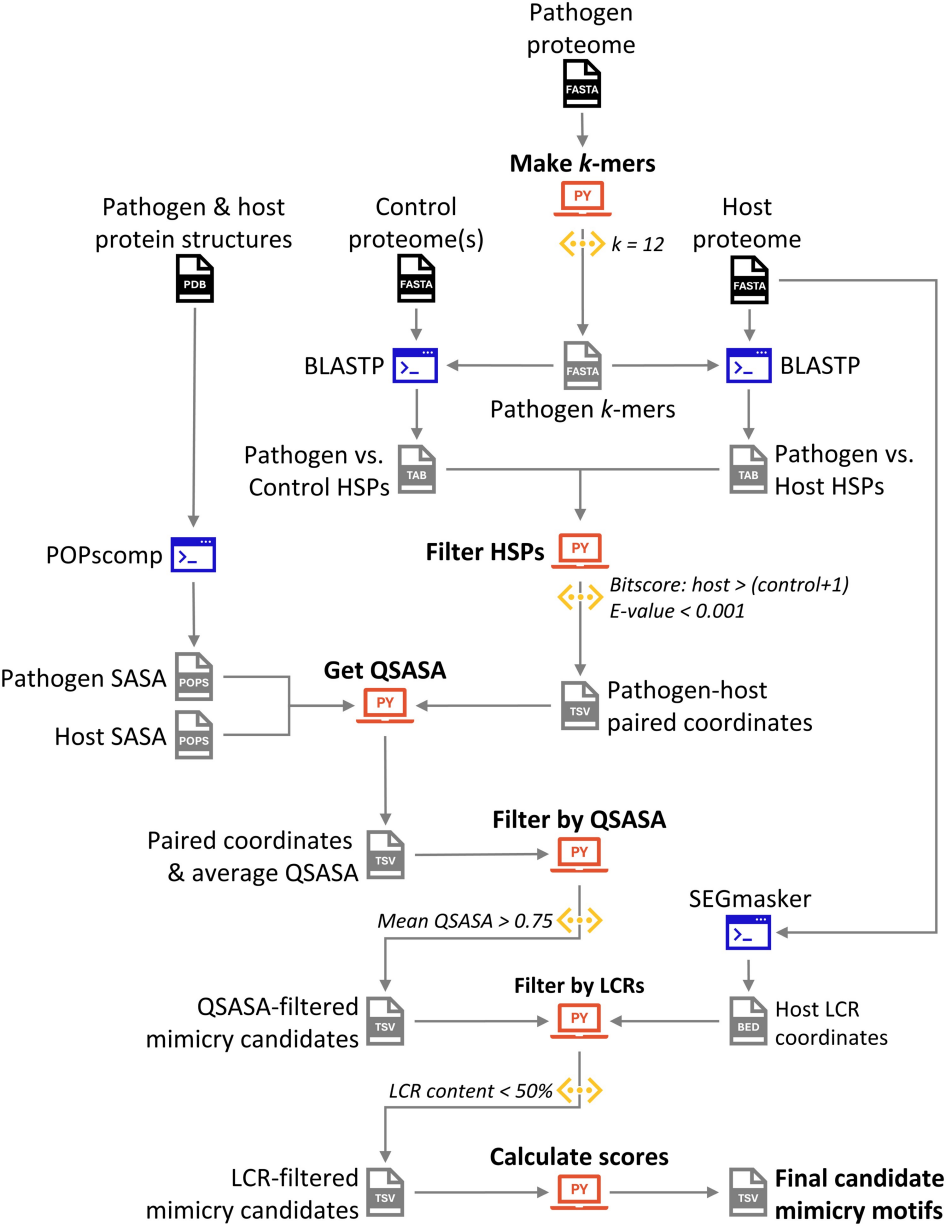
Workflow for the mimicDetector pipeline with suggested thresholds.

### 2.2 Alignment recall

BLASTP consistently outperformed other alignment tools for both identical and similar alignments (Figs S2 and S3, available as supplementary data at *Bioinformatics* online). For exact matches, the PAM30 substitution matrix outperformed BLOSUM62 when *k *≤ 9 and was equivalent at *k *≥ 10. Reducing *wordsize* to 2 improved recall for *k *= 5, where seeding was a limiting factor. For similar-hit alignments, BLASTP again led, though performance varied. PAM30 outperformed BLOSUM62 when: (i) *k *≤ 9; (ii) *k *≥ 10 with few mismatches. In contrast, BLOSUM62 was slightly better PAM30 for the longer *k*-mers. Reducing *wordsize* helped with PAM30-scored alignments, but had little effect on BLOSUM62-scored alignments.

TOPAZ performed well for identical *k*-mer alignments. However, it was excluded due to a mismatch-counting error and weaker similar-hit performance. DIAMOND and PHMMER underperformed compared to BLASTP across all tests. Glam2Scan showed perfect recall for identical-hits but was excluded due to excessive runtimes (Fig. S4, available as supplementary data at *Bioinformatics* online).

### 2.3 Proteome coverage

Beyond recall, we assessed how much of the proteome was matched by the query *k*-mers. We consider it desirable to find a higher coverage of the target proteome, so reducing the likelihood of excluding potentially relevant motifs early in the pipeline. Merging overlapping alignments, we found that BLASTP with PAM30 and *wordsize* = 2 provided the highest coverage, plateauing at *k* = 12 (Fig. S5, available as supplementary data at *Bioinformatics* online).

### 2.4 False discovery rate

We estimated the empirical false discovery rate (FDR) using a target–decoy approach, with the human proteome as the target and shuffled human sequences as the decoy:


(1) $$\mathrm{FDR}=c\cdot (N_{\mathrm{decoy}}/N_{\mathrm{target}})$$

where *N*_target_ is the number of hits to the target database, *N*_decoy_ is the number of hits to the decoy database, and *c* is a correction factor for the number of unique peptides (UP) in the target and decoy databases (Käll *et al.* 2008, Lee *et al.* 2021):


(2) $$c=1/({UP}_{\mathrm{decoy}}/{UP}_{\mathrm{target}})$$

We calculated FDR for runs of mimicDetector for combinations of threshold value ranges for bitscore difference (0≤*b *≤ 3), minimum E-value (range 0.05≥*e *≥ 0.0005) and minimum solvent accessibility (*q *= 0.50 or 0.75) for the three aforementioned species. We found that combinations of 0≤*b *≤ 2, 0.01≥*e *≥ 0.001, and *q *= 0.75 produced FDR < 0.05 for all three species but runs with *b *= 1 and *e *= 0.001 consistently had the lowest FDR, regardless of dataset (Fig. S6, available as supplementary data at *Bioinformatics* online).

### 2.5 Homologue removal

The original pipeline filtered full-length pathogen proteins with significant alignments to host proteins, presumably to reduce the number of *k*-mers to consider in later searches. However, this may exclude proteins due to a shared promiscuous domain or biologically relevant mimics contains within homologous genes that duplicated and undergone subfunctionalization (Pastrana *et al.* 1998, Armijos-Jaramillo *et al.* 2021, Huang *et al.* 2022). We found that removing this step led to a small increase in the number of candidate motifs in *P. falciparum* and a marked increase in helminth species, which are discussed in the next section (Fig. S7, available as supplementary data at *Bioinformatics* online).

## 3 Implementation

mimicDetector integrates optimized alignment parameters and refined filtering strategies into a streamlined, modular pipeline (Fig. 1). We selected the BLASTP search algorithm, with the PAM30 substitution matrix and *wordsize *= 2 (Table S3, available as supplementary data at *Bioinformatics* online). The default *k*-mer length is 12 amino acids. A further change to early versions is the use of bitscore, rather than percent identity, to compare *k*-mer alignments.

Users provide proteomes for the pathogen, host, and negative control species (typically non-pathogen) in FASTA format. The pathogen proteome is fragmented into overlapping *k*-mers, which serve as queries for ungapped local-local alignments.

Post-alignment, mimicDetector applies a series of biologically motivated filters. To achieve an FDR < 0.05 (see section 2.4), we retain alignments where E-value is ≤0.001 and the pathogen-host alignment bitscore exceeds that of the best pathogen-control alignment by at least one bit. Overlapping *k*-mer alignments are merged into a single preliminary mimicry candidate. Finally, hits with a mean solvent accessible surface area >75%, via POPSCOMP, are kept, and hits where >50% of the sequence is low-complexity, via Segmasker, are removed (Wootton and Federhen 1996, Kleinjung and Fraternali 2005).

The final output includes a ranked list of predicted mimicry candidates, which includes pathogen and host protein sequences with positional information and recalculated alignment scores and E-values. The pipeline and its accompanying documentation are freely available on GitHub.

## 4 Results and discussion

We applied mimicDetector to 17 eukaryotic pathogen proteomes to identify protein-level mimicry targeting the human proteome (Table S1, available as supplementary data at *Bioinformatics* online). The updated pipeline consistently identified more mimicry candidates than our previous approach, particularly when homologous proteins were retained and evaluated at the *k*-mer level.

In *Leishmania infantum*, we identified two notable mimics. The first protein, LINF_180015000 (UniProt: A4HXX0), shares a motif with CD244 (2B4; UniProt: Q9BZW8), an immune checkpoint receptor expressed on natural killer cells (De Freitas *et al.* 2021, Sanz *et al.* 2021). The second protein, LINF_310020500 (UniProt: A4I6R4), shared a motif with Reticulon-4B (Nogo-B; UniProt: Q9NQC3), a modulator of leukocyte migration and TLR9 trafficking, which has been implicated in Leishmania-driven changes to macrophage behavior (Kimura *et al.* 2015, Bhattacharya *et al.* 2023).

In helminths (parasitic worms), mimicDetector uncovered possible mimicry of complement components. Four *Schistosoma mansoni* proteins had similarity to C1qA and four nematode species matched C1qB (Table S4, available as supplementary data at *Bioinformatics* online). Many helminth species, including those mentioned here, secrete a calreticulin homologue which inhibits classical activation of the complement pathway through interaction with complement protein C1q (Floudas *et al.* 2017, Yadav *et al.* 2021, Esperante *et al.* 2023). Functional assays are planned to examine the potential interactions suggested by mimicDetector.

Finally, we note that the search algorithms tested here were not originally designed for our specific goal. For example, the long runtimes for Glam2Scan were in part due to formatting the data appropriately for each search. Future mimicry detection may benefit from using underlying algorithms, such as Waterman-Eggert (Waterman and Eggert 1987).

## Supplementary Material

btag012_Supplementary_Data

## Data Availability

No new data were generated or analysed in support of this research. The data analysed for this article are available from Uniprot using the accession codes in Table S1 of the supplementary material.
