# Supplementary material for: mimicDetector: a pipeline for protein motif mimicry detection in host-pathogen interactions
Source: Bioinformatics. 2026 Jan 12;42(2):btag012. doi: 10.1093/bioinformatics/btag012 (PMC12881831; doi:10.1093/bioinformatics/btag012)
Supplement: btag012_Supplementary_Data [file btag012_supplementary_data.zip › SOM_Figures_20260108.pdf]

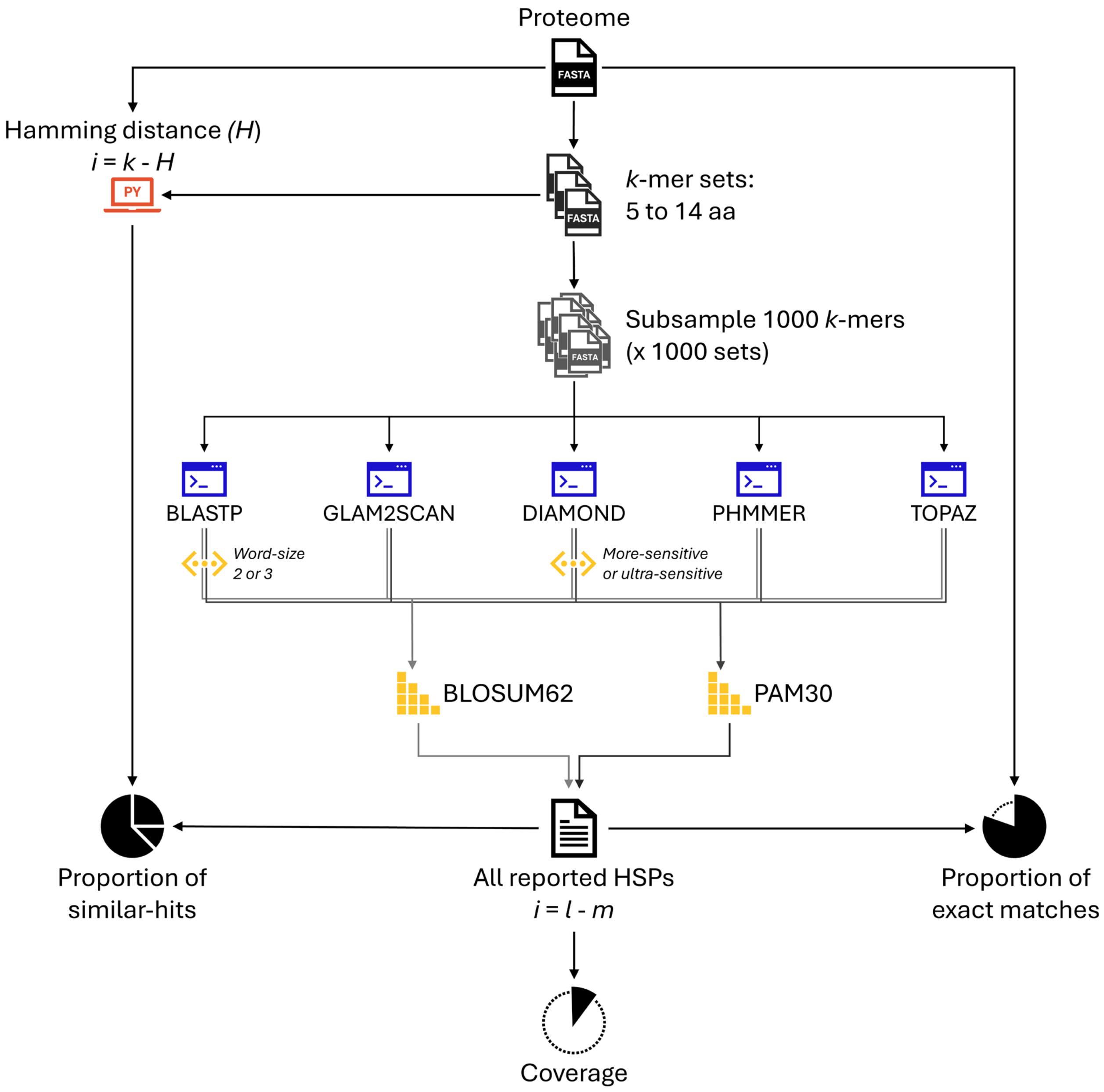

Figure S1: Algorithm-parameter sets assessment workflow. Yellow icons indicate steps where more than one parameter was tested. True positives were determined through calculation of Hamming distance for each query *k*-mer: number of identical residues (i) = *k*-mer size (k) - Hamming distance (H). Number of identical residues for each tool/parameter combination were calculated for all reported HSPs: i = reported alignment length (l) - mismatches (m) for each size.

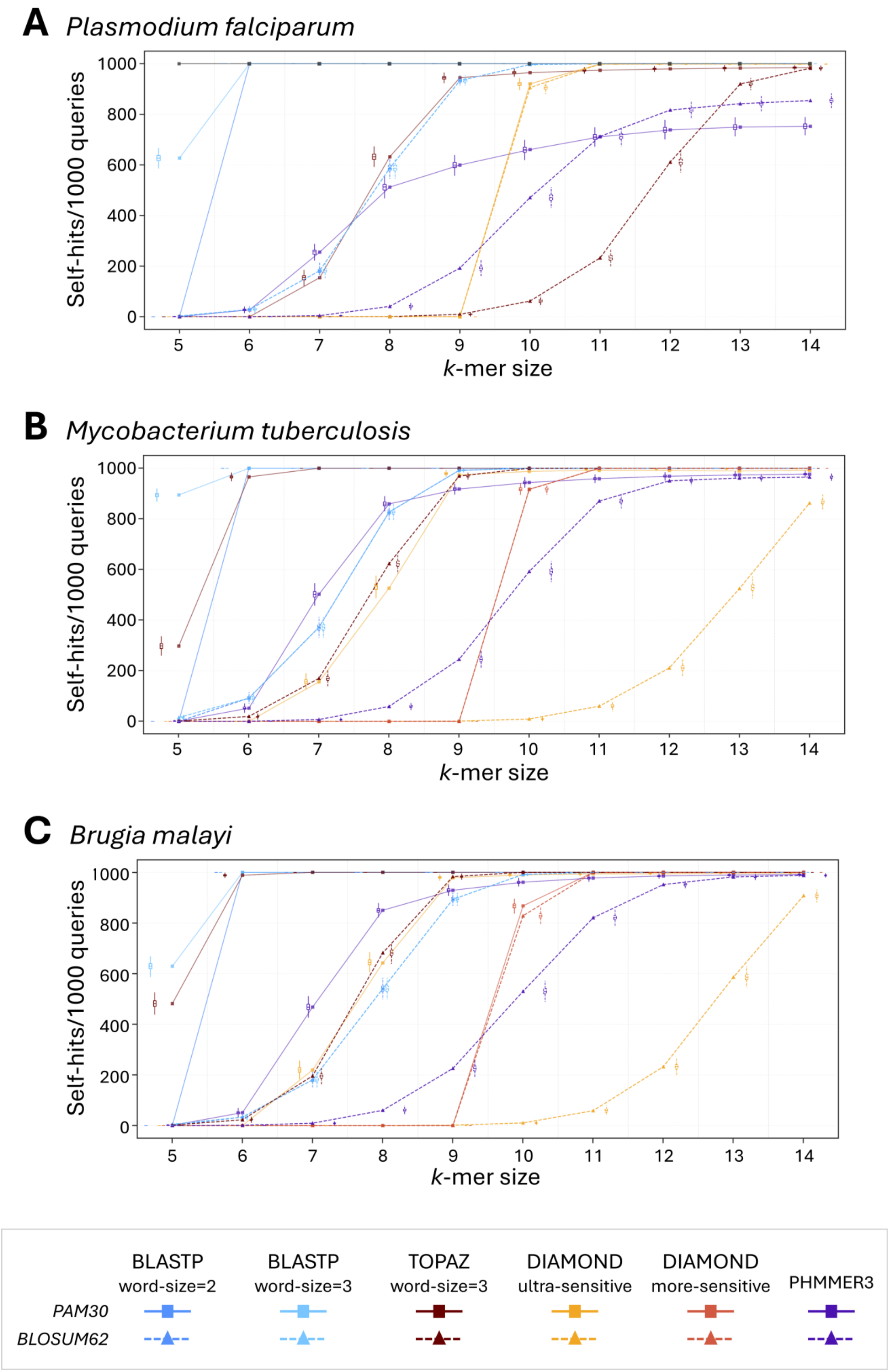

Figure S2: Recall of identical hits when querying 1000 *k*-mers against the full protein database for each tool/parameter combination and *k*-mer size.

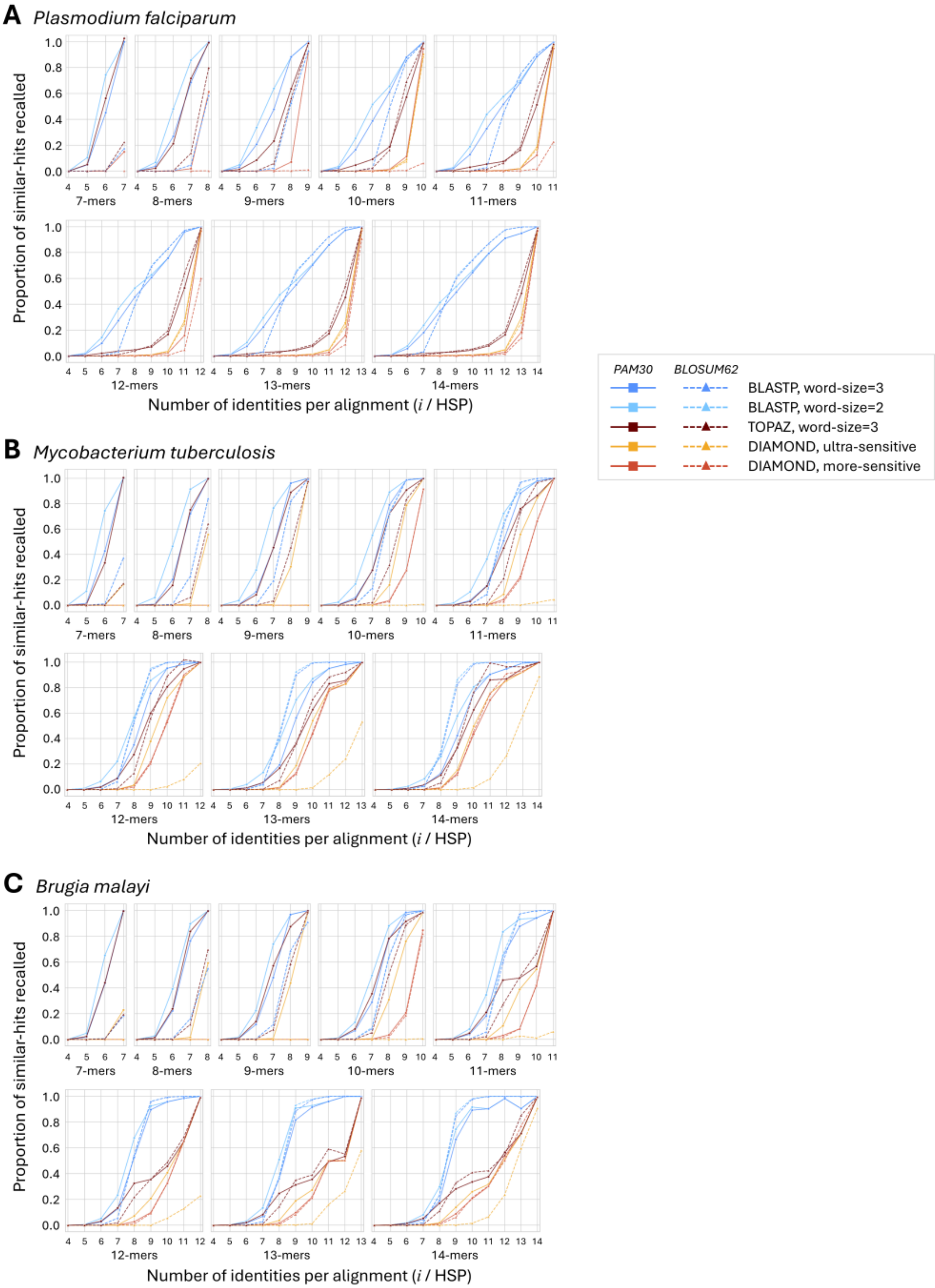

Figure S3: Recall of similar hits when querying 1000 *k*-mers against the full protein database for each tool/parameter combination and *k*-mer size. Similar-hits were calculated by the proportion of true positives with *i* identities as depicted in Fig S1.

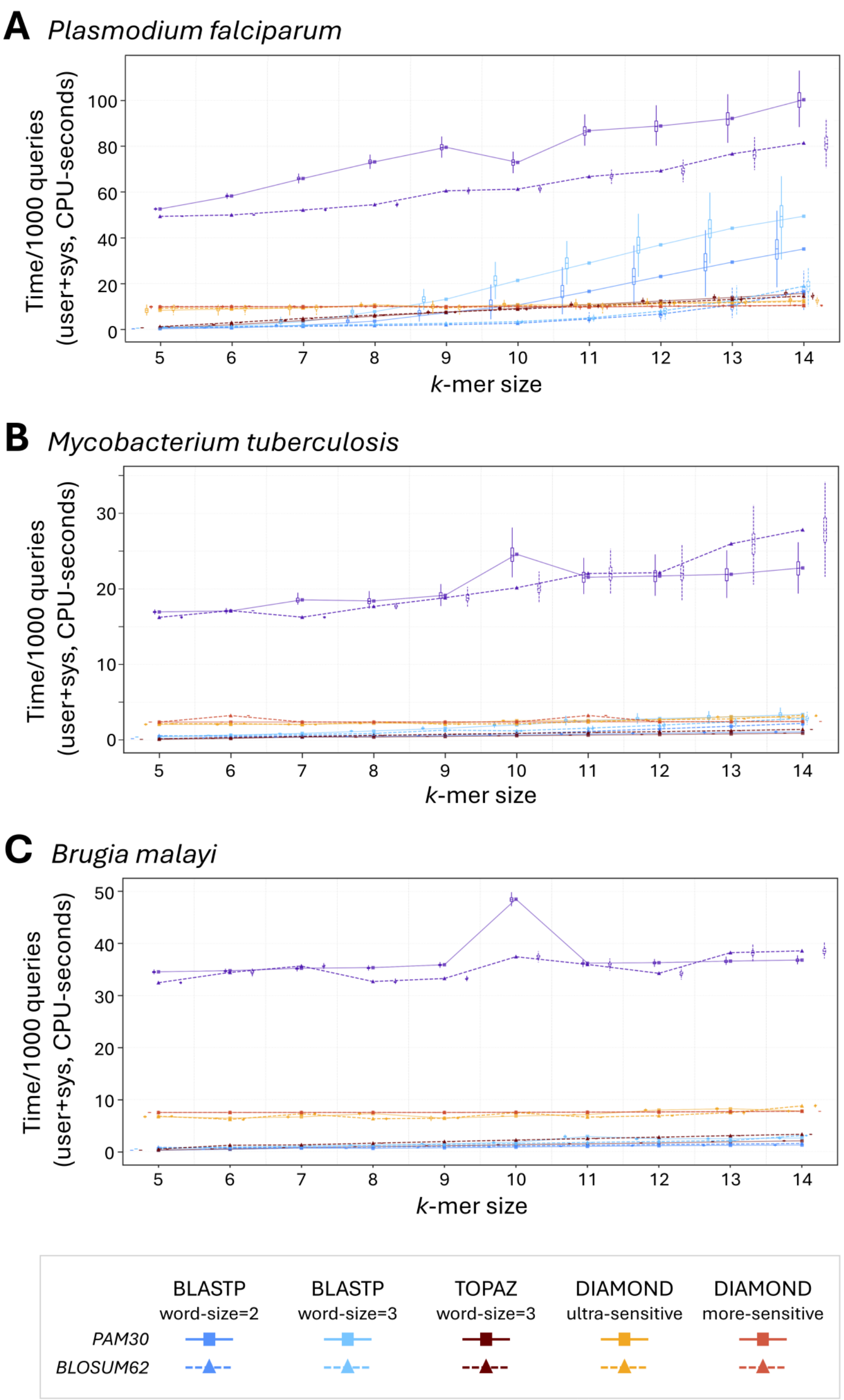

Figure S4: Runtime. Time (user + sys CPU time (seconds) used in executing the process) to query 1000 *k*-mers against the full protein database for each tool/parameter combination and *k*-mer size.

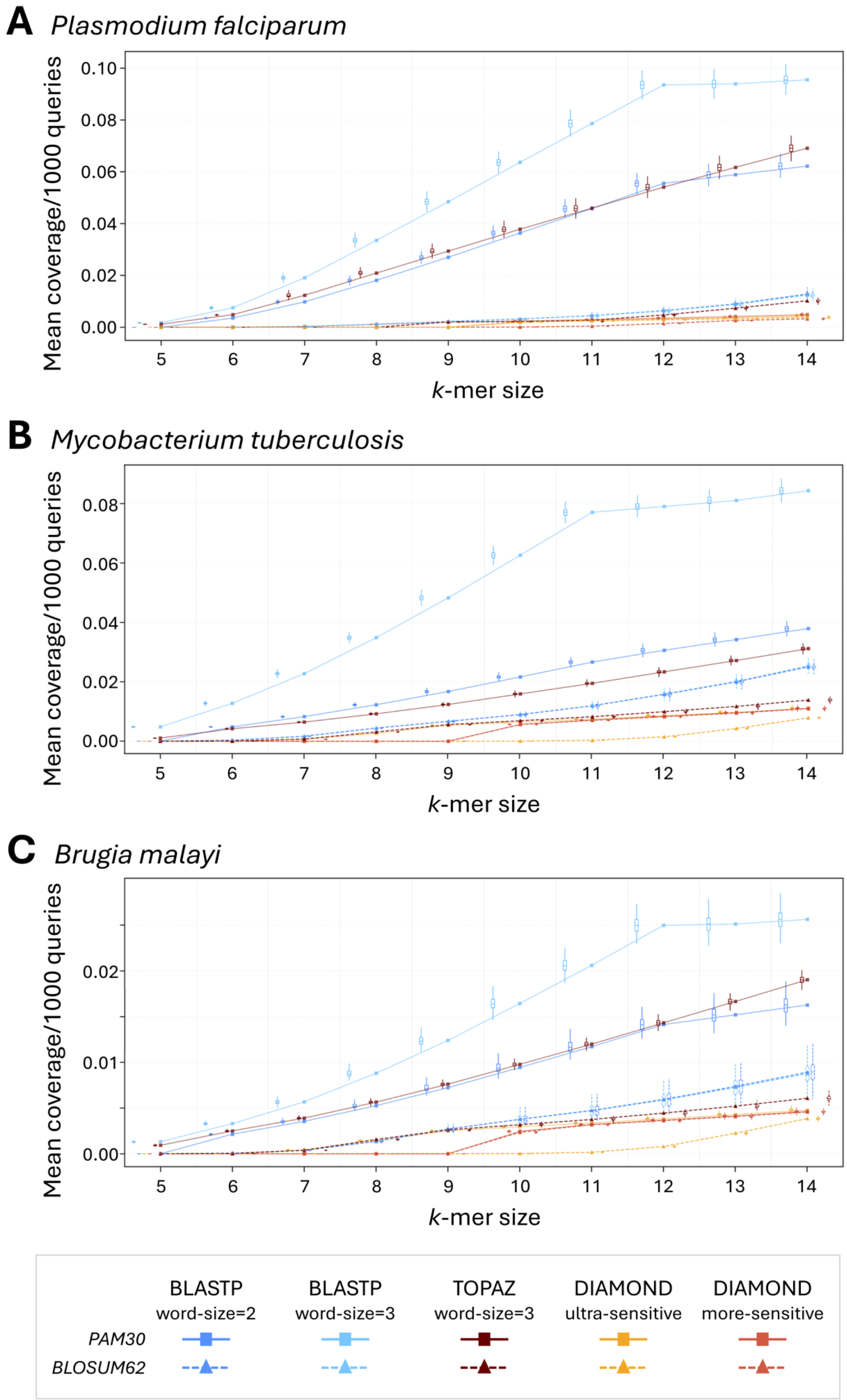

Figure S5: Mean coverage of proteome identified by each tool/parameter combination for each *k*-mer size after removing LCRs (query *k*-mers < 50% content).

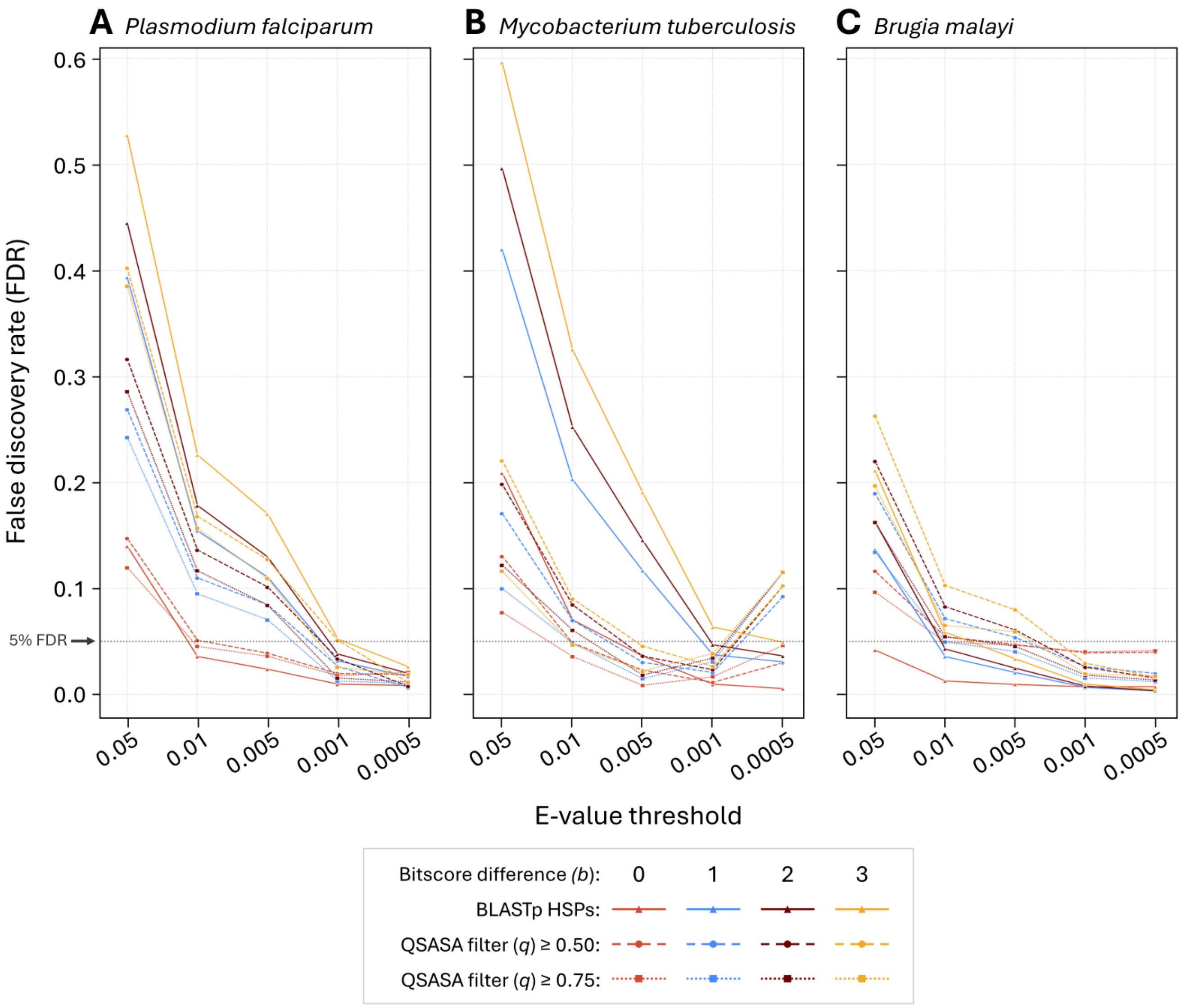

Figure S6: Estimated false discovery rates for $k = 12$ calculated using a target-decoy approach for bitscore differences (b) ranging from 0 to 3 before (HSPs, solid lines), after filtering for mean QSASA (q) ≥ 0.50 (dashed lines), and $q \geq 0.75$ (dotted lines) at E-value thresholds (e) ranging from 0.05 to 0.0005.

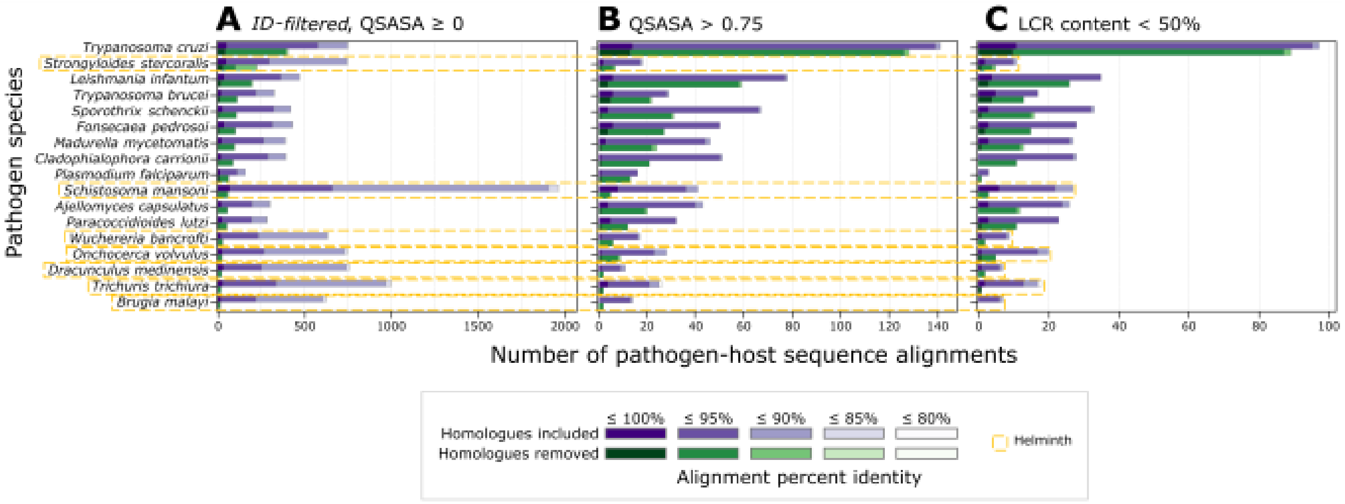

Figure S7: Number of mimicry candidates identified for individual species with and without initial homologue filtering. Number of mimicry candidates from runs with homologues included (top bar, purple) and with homologues excluded (bottom bar, green) Helminth species are indicated by dashed boxes to highlight the greater number candidates retained at each filtering stage: A) after BLASTP runs with HSP identity-based filtering, B) after filtering for solvent accessibility, and C) after filtering for low complexity regions.
